# Mitochondrial DNA: A New Predictor of Diabetic Kidney Disease

**DOI:** 10.1155/2020/3650937

**Published:** 2020-07-15

**Authors:** Yajing Huang, Jingwei Chi, Fanxiang Wei, Yue Zhou, Yihai Cao, Yangang Wang

**Affiliations:** ^1^Department of Endocrinology, The Affiliated Hospital of Medical College of Qingdao University, Qingdao 266003, China; ^2^Department of Microbiology, Tumor and Cell Biology, Karolinska Institutet, Stockholm 171 77, Sweden

## Abstract

Diabetic kidney disease (DKD) is a common cause of end-stage renal disease, and diagnosis and treatment in time can help delay its progress. At present, there are more and more studies on the pathogenesis of DKD; mitochondrial dysfunction plays an important role in DKD. The occurrence and development of DKD is closely related to epigenetic changes and the interaction between mtDNA, ROS, inflammatory factors, and endothelial damage, which continuously aggravates kidney. The change of mtDNA is both the cause of DKD and the result of DKD. It is of great significance to incorporate the change of mtDNA into the monitoring of patients with diabetes. Existing evidence indicates that changes in mtDNA copy number in blood and urine reflect mitochondrial dysfunction and the severity of DKD. However, large-scale, long-term follow-up clinical trials are still needed to determine the threshold range. By the time, mitochondrial-targeted antioxidants will become a new method for the treatment of DKD and other diabetic complications; mtDNA also can be a therapeutic target for them.

## 1. Introduction

Worldwide, the incidence of diabetes mellitus (DM) has increased rapidly and has affected more than 380 million people, of which China has the highest rate of diabetes patients, approximately 98.4 million [1]. Diabetic kidney disease (DKD) is the most serious microvascular disease in diabetes. In developed countries, DKD is the main cause of end-stage renal disease, accounting for about 44.5% of cases [2]. DKD is defined as a syndrome that is characterized by leakage of protein (in particular albumin), metabolites, and ions into the urine, changes in the glomerular filtration rate (GFR), and an increased risk of cardiovascular disease (CVD) and stroke [3]. The progressive exacerbation of DKD is manifested by the continued exacerbation of albuminuria and the gradual decline of glomerular filtration rate, which eventually leads to complete loss of renal function [4]. Before the emergence of clinical albuminuria (Mogensen stage III), there will be more insidious disease stages, during which the glomerular filtration rate is normal or highly filtered (Mogensen stages I and II), but there are no simple detection methods for diagnosis at present. Tight glucose control and antihypertensive drugs are now recognized as the main means of preventing early clinical DKD [4, 5]. New antidiabetic drugs have renal protection while controlling blood glucose. For example, sodium glucose transporter 2 (SGLT2) inhibitors can reverse the hemodynamic changes observed in the afferent and efferent arteries and reduce glomerulosclerosis [6]. Angiotensin-converting enzyme inhibitors (ACEI) and angiotensin II receptor blockers (ARB) have become the first-line drugs for patient with DKD to control blood pressure and improve the prognosis of DKD. But these drugs can only delay the development of DKD, but cannot reverse DKD [4].

The main histological features of the disease are glomerular basement membrane (GBM) thickening, glomerular mesangial expansion (GME), podocyte loss, glomerular sclerosis (GS), and progressive general fibrosis [7, 8]. Renal fibrosis is the ultimate pathological change of DKD. It is caused by a variety of mechanisms including renal hemodynamic changes, oxidative stress, inflammatory response, hypoxia, and activation of the renin-angiotensin-aldosterone system (RAAS) [9, 10]. The kidney is a high metabolic organ; increasing evidence indicates that reactive oxygen species (ROS) overproduction is a common cause of kidney metabolic changes and hemodynamic changes [11]. Increased mitochondrial superoxide can lead to hyperglycemia, mitochondrial DNA (mtDNA) damage, and activation of apoptotic pathways [12]. Changes in mtDNA are closely related to DKD [3, 13]. In the prediabetes period, fasting blood glucose and HbA1C had not changed significantly. But mtDNA NADH-6 (ND6) and D-loop had been methylated in these patients, and ND6 and D-loop methylation were closely related to insulin resistance [14]. Changes in mtDNA preceded DKD's bioenergy dysfunction and induced inflammatory response [15]. mtDNA changes were observed in tissue, blood, and urine. Mitochondrial protection has become a new target for the treatment of DKD [16]. We believe that monitoring changes in mtDNA can predict the occurrence of DKD, and it also can be a target for early intervention to delay the progress of DKD.

## 2. mtDNA Features

Mitochondrion is a unique organelle that plays an important role in energy production, programmed cell death, calcium homeostasis, and synthesis of lipids, amino acids, and heme. Unlike the nuclear genome, mitochondria have their own genome, although the genome encodes only 13 peptides involved in oxidative phosphorylation [17]. However, these peptides are the basic components involved in the oxidative phosphorylation chain; they are essential for ATP production during respiration [17]. The double-stranded structure of mtDNA consists of a guanine-rich heavy (*H*) strand and a light (*L*) strand [15]. mtDNA is particularly vulnerable to damage, especially through the generation of oxidative lesions. First, mtDNA resides in close proximity to the site of ROS production in the mitochondrial membrane. Second, mtDNA replication proceeds via an asymmetric route, resulting in parts of the heavy strand existing as single-stranded structures for extended periods of time; this can lead to the spontaneous deamination of nucleotides [18]. Compared with genomic DNA, lower concentrations of ROS can cause damage to mtDNA, and under long-term oxidative stress, mtDNA damage is repaired more slowly than genomic DNA [19]. Impaired mtDNA and disrupted mitochondrial genome integrity play important roles in the development of severe early-onset and chronic aging-related diseases [19]. It is becoming increasingly clear that long-term, tiny mtDNA damage is not only related to the aging process, but may also be closely related to diabetes and its complications [20].

During early DKD, the ATP demand of kidney cells increased due to ultrafiltration, and increased ATP synthesis was caused by glycolysis. Chronic hyperglycemia disrupts the bioenergetic balance between the kidney's energy requirements and the supply of metabolic fuel, which is conducive to the production of ROS [21]. mtDNA in the blood can cross the kidney barrier and is excreted in the urine. Therefore, the detection of mtDNA in blood and urine can be used as a metabolic marker of early DKD (Table 1).

## 3. Change of mtDNA in DKD

### 3.1. Change of mtDNA in Tissues

Changes in mtDNA in tissues were found in pancreatic islet *β* cells [26], skeletal muscle cells [27], animal models of diabetic retinopathy [28], and models of diabetic peripheral neuropathy [29] in diabetic patients. These include mitochondria morphological damage and decreased mtDNA copy number. Besides, oxidative mtDNA damage and mtDNA deletion were also observed in streptozotocin-induced diabetic rats [30]. In DKD mice, damage to mtDNA was localized to glomerular endothelial cells [12]. While in patients with DKD, mitochondrial fragmentation was specifically presented in tubules, but not in podocytes of DKD patients [25]. This contradicts the findings in animal models. The evolution of renal pathological changes in DKD mouse models is different from DKD patients [31]. When exploring the therapeutic targets of DKD, it is important to choose the appropriate mouse model.

### 3.2. Change of mtDNA in Blood

Sharma first reported that the copy number of mtDNA could be used as a new biomarker for DKD [13]. High glucose conditions downregulated the mtDNA content in mouse podocytes and promoted the release of mtDNA extracellularly. Due to the damage to mitochondrial biology, the amount of mtDNA in tissues (such as heart, liver, pancreas, and kidney) in the DM model decreased, while the amount of mtDNA in peripheral blood increased in patients with DM. Early increase in mtDNA copy number is closely related to insulin resistance and increased inflammatory factors [22, 24, 32]. Al-Kafaji et al. observed similar reductions in mtDNA copy number in peripheral blood in clinical trials. Reduced mtDNA copy number was negatively correlated with albuminuria and risk factors for DKD and positively correlated with estimated glomerular filtration rate [23]. In the early stage of DM, the mtDNA copy number in peripheral blood increased, and in patients with DKD, the mtDNA copy number in peripheral blood decreased. This indicates that changes in mtDNA copy number in peripheral blood can predict the occurrence of DKD. Regular monitoring of mtDNA changes in peripheral blood in DM patients can be used as a predictor of DKD and reflect the severity of DKD.

### 3.3. Change of mtDNA in Urine

mtDNA in the blood can cross the kidney barrier and is excreted in the urine. Therefore, mtDNA in urine can also be used as a metabolic marker of DKD. Studies by Wei et al. showed that mtDNA levels in urine supernatants increased. It was associated with a decrease in mtDNA in the kidney (*r* = −0.453, *P*=0.012) and was positively correlated with the severity of renal interstitial fibrosis (*r* = 0.300, *P*=0.005) [33]. In the DKD model, the essential mitochondrial genes of glomerular cells were significantly downregulated, and mitochondrial damage was increased. The damage marker 8-oxoguanine (8-oxodG) could be secreted into urine. In the early stage, mtDNA damage was limited to glomerular endothelial cells and accumulated over time, increasing urine secretion of 8-oxodG. The level of 8-oxodG is related to the rate of progression of renal disease [12]. Cao et al. found that urine mtDNA/creatinine ratios increased in urine samples from patients with DM, especially those with DKD. At early microalbuminuria, urine changes occurred before changes in blood mtDNA [24]. Urine mtDNA changes occur earlier and are closely related to renal fibrosis, reflecting the progress of DKD.

Urine as an easily obtained and noninvasive sample source has an advantage over tissue and blood. Therefore, urine mtDNA is a good indicator for diagnosing DKD and is a prognostic indicator of DKD [34].

## 4. Mitochondrial Oxidative Stress and mtDNA in DKD

### 4.1. Mitochondrial Oxidative Stress in DKD

Kidney is the organ with the second highest oxygen consumption in our body; it is distinctly sensitive to mitochondrial dysfunction. Mitochondrial dysfunction contributes to the progression of DKD [35]. Mitochondrial dysfunction is characterized by the increase of mitochondrial ROS production, mitochondrial permeability transition (MPT) pore opening, and apoptosis [36]. Mitochondria are extremely vulnerable to hyperglycemia. Because mtDNA lacks protection and repair mechanisms, it can lead to a decrease in mtDNA [37]. The ATP content of the kidney in the DM mouse model decreased, and the copy number of mtDNA decreased, indicating that mitochondrial biological dysfunction and biogenesis dysfunction exist in the kidney [16]. The mechanisms of mitochondrial oxidative stress causing kidney damage include the following. (1) High glucose activates the JNK-CaMKII-Fis1 pathway, causing mitochondrial fragmentation, increased ROS, and JNK activation enhances podocyte apoptosis and renal tubular cell damage in mice [38]. (2) Mitochondrial dysfunction inhibits the AMPK-SIRT-1-PGC-1*α* pathway and downregulates podocyte autophagy, and podocyte damage continues to accumulate, resulting in increased albuminuria [16, 39]. (3) Excessive ROS induces the production of TGF-*β*, downregulates NO, and causes renal fibrosis and endothelial cell damage [40].

### 4.2. Mitochondrial Electron Transport Chain and mtDNA

ROS are produced during the normal operation of the mitochondrial electron transport chain (mtETC). When mtETC fails, ROS are overproduced. In kidney cells, hyperglycemia causes an increase in protein kinase C (PKC). The increase in PKC induces endothelial nitric oxide synthase (eNOS) production and increases the utilization of nitric oxide (NO) in the early stages of DKD [41]. Increased nitric oxide (NO) contributes to the activation of vascular endothelial growth factor (VEGF), which leads to endothelial dysfunction [42, 43]. When endothelial cell dysfunction occurs, endothelial cell mitochondrial function is inhibited and ROS production is increased. Excessive ROS inhibit mammalian target of rapamycin 1 (mTORC1) and AMP-activated protein kinase (AMPK) and affect the activation of peroxisome proliferator-activated receptor-*γ* coactivator-1*α* (PGC-1*α*). As a major regulator of mitochondrial biogenesis, PGC-1*α* is beneficial to mitochondrial biogenesis and the reparation of oxidative stress caused by ROS. The downregulation of PGC-1*α* reduces the amount of mtDNA. Impaired gene expression leads to impaired synthesis of mtETC protein, accumulation of ROS, and continuous accumulation of damage to mtDNA, causing kidney damage [44, 45]. The DKD model also found that microtubular autophagic vacuoles and mitochondrial fragmentation were reduced under the microscope. The accumulation of these broken mitochondria also caused excessive production of ROS and aggravated oxidative stress [46].

### 4.3. Vicious Circle between ROS, mtDNA, and DKD

The mtDNA changes caused by mitochondrial oxidative stress aggravate the vicious cycle of DKD. The components are as follows: (1) high glucose leads to increased ATP demand, resulting in mtETC producing more ROS; (2) excess ROS destroys phospholipids, proteins, and nucleic acids; (3) ROS inhibits mitochondrial germinal growth by downregulating PGC-1*α*, and the amount of mtDNA decreases; (4) decreased synthesis of mtDNA-encoded subunits impairs the electron transport system and further augments the generation of superoxide promoting damage to mtDNA [47]; (5) ROS and damaged mtDNA continue to accumulate, which leads to podocyte and renal tubular cell damage, and aggravate by renal fibrosis.

## 5. Inflammation and mtDNA in DKD

mtDNA, when released extracellularly, could act as a damage‐associated molecular pattern (DAMP) agent and cause inflammation [48]. Cao et al. pumped excess mtDNA into DM mice. Under diabetes, more mtDNA filtered by the kidney is associated with chronic sterile inflammation of the kidney [24]. The circulating cell-free mtDNA (ccf-mtDNA) can also activate the inflammasome (NLRP3). NLRP3 initiates inflammatory cascades that lead to activation of caspase-1 and the production of IL-1*β* and IL-18 through the TLR-NF-*κ*B pathway [49, 50]. The NLRP3 inflammasome links sensing of metabolic stress in the diabetic kidney disease [51]. ccf-mtDNA also acts as a critical signaling molecule in chronic inflammation via absent in melanoma 2 (AIM2) inflammasome activation. AIM2 also can promote caspase-1 activation and IL-1*β* and IL-18 secretion in macrophages [52]. Studies have reported that overexpression of IL-18 increased neutrophil infiltration and worsened kidney damage [53]. IL-1*β* and IL-18 impair podocyte and endothelial function independent of inflammatory cell recruitment [54]. Under long-term hyperglycemia, the kidney's self-repairing ability is impaired, and continuous inflammation continues to aggravate kidney damage.

## 6. Mitochondrial Antioxidants and mtDNA

Mitochondria-targeted antioxidant mitoTEMPO has a significant effect on reducing the accumulation of 8-oxoG in glomerular endothelial cells, reducing the level of 8-oxodG in urine, and has protective effects on endothelial cells and podocytes [12]. MitoQ protects against hyperglycemia-induced oxidative injury in tubular cells to maintain mitochondrial quality and also partially mediates mitophagy via Nrf2/PINK [55]. However, current clinical trials have shown that the efficacy of antioxidants (alpha lipoic acid, vitamin C, coenzyme Q10, mitoQ, etc.) on diabetic complications is not consistent [47, 56, 57]. Mitochondria inner membrane is highly impermeable, and this could decrease the efficacy of the supplements since they cannot cross the membrane and reach the targets [47].

In addition, in animal models of diabetes, Chinese herbal extracts such as Bombax ceiba L. leaves and isoorientin have also been shown to have a role in kidney protection, reducing mitochondrial oxidative stress, reducing ROS accumulation, and protecting mtDNA [58, 59]. Salidroside and grape seed procyanidin B2 play a beneficial role against DKD in mice, which probably via AMPK/Sirt1/PGC-1*α*-mediated mitochondrial biogenesis. It also enhanced mtDNA copy and electron transport chain proteins [60, 61]. Chinese herbal extracts have achieved good results in the treatment of DKD, especially by improving mitochondrial oxidative stress. The safety and toxicity of traditional Chinese medicine still need to be verified with more rigorous experiments.

## 7. Discussion

DKD is a serious complication of diabetes. Studies have shown that early intensive glycemic control has certain benefits in delaying complications of diabetes [62]. In prediabetes, the epigenetics of mtDNA has begun to change. Before the occurrence of diabetic retinopathy, mtDNA methylation increased, and during the period of diabetic retinopathy, mtDNA methylation decreased [63]. This shows that mtDNA is very important in the occurrence and development of diabetes and its complications. Including mtDNA changes in the monitoring of diabetic patients is of great significance for long-term prognosis.

**Figure 1:**
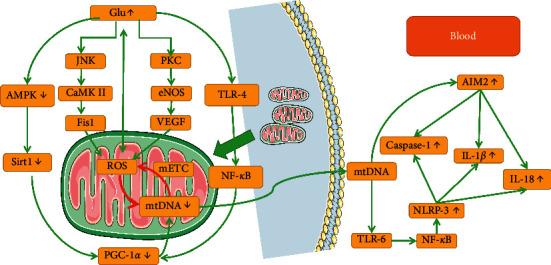
mtDNA, mitochondrial oxygen stress, and inflammation in DKD.

In the early stage of DKD, the kidney showed a high filtration state; the ATP demand of kidney cells increased, causing mitochondrial oxidative stress and increased ROS production through downregulation of PGC-1*α* and damage to mtDNA [45]. These mtDNA damages were first verified in glomerular endothelial cells, and secretion of 8-oxodG, a marker of mtDNA damage in urine, was increased [12]. Damaged endothelial cells reduce mitochondrial function, increase superoxide production, and damage mtDNA. Changes in the endothelial glycocalyx openings induce apoptosis of endothelial cells and cause impaired glomerular filtration. Eventually, albuminuria appears and renal function gradually decreases [64, 65]. The early state of high glucose also induces the transfer of mtDNA into peripheral blood circulation. As a DAMP agent, ccf-mtDNA triggers a cascade of inflammation by activating inflammasomes (NLRP-3 and AIM2) and induces chronic sterile inflammation in the kidney (Figure 1).

Although antioxidants can reduce oxidative stress in kidney tissue, they cannot completely reverse mtDNA changes, such as mtDNA deletion [30]. This suggests that oxidative stress is only one of the pathways for mtDNA damage. Exploring other damage pathways of mtDNA under high glucose conditions and using this as a target to intervene may become a new therapeutic approach for DKD.

Therefore, we believe that in the occurrence and development of DKD, mtDNA changes are both the cause and the result. It is of great significance to incorporate the changes in mtDNA into the monitoring of DM patients: (1) the methylation level of mtDNA can be used as a predictive index for DM high-risk groups (obesity or family history of DM); (2) the ccf-mtDNA copy number can be used as a predictor of DKD in the DM population with obesity or family history of DM; (3) ccf-mtDNA can also reflect chronic sterile inflammation in patients with DM or DKD; (4) mtDNA copy number of urine can be used as a diagnostic indicator of DKD and monitoring indicators of DKD progress; (5) ccf-mtDNA and urine mtDNA can be used as an indicator to improve mitochondrial oxidative stress by targeting mitochondrial antioxidants and Chinese herbal extracts; (6) exploring other damage pathways of mtDNA under high glucose conditions may become a new therapeutic approach for DKD.

Although in animal models and some small-scale clinical trials, it has been shown that changes in mtDNA are related to the progress of DKD [16, 23]. At present, large-scale clinical trials are still needed to explore the threshold of mtDNA copy number for DKD diagnosis and the relationship between mtDNA and DKD prognosis. Besides, mtDNA copy number detection is expensive, which has become an important reason for limiting mtDNA as a routine monitoring of DKD. Therefore, it is necessary to develop a simple and inexpensive detection method to determine the mtDNA copy number.

## Figures and Tables

**Table 1:** Clinical trial about mtDNA changes in DKD or T2DM.

Author	Year	Samples (*n*)	Treatment group	Control group	Observation index	*P* value
Sharma et al. [13]	2013	32	DKD	Healthy	Urinary mtDNA	≤0.01
Deng et al. [22]	2019	244	T2DM	Healthy	Plasma mtDNA	<0.001
Al-Kafaji et al. [23]	2018	100	DKD	T2DN	Plasma mtDNA	<0.01
Al-Kafaji et al. [23]	2018	100	DKD	Healthy	Plasma mtDNA	<0.01
Cao et al. [24]	2018	77	DKD	Healthy	Plasma mtDNA	0.012
Cao et al. [24]	2018	77	DKD	Healthy	Urinary mtDNA/Cr ratio	<0.001
Jiang et al. [25]	2019	108	DKD	T2DM	Plasma mtDNA	0.036

## Abbreviations

Glu: Glucose
ROS: Reactive oxygen species
mtDNA: Mitochondrial DNA
mtETC: Mitochondrial electron transport chain
AMPK: Adenosine 5-monophosphate- (AMP-) activated protein kinase
Sirt1: Sirtuin1
PGC-1*α*: Peroxisome proliferator-activated receptor-*γ* coactivator-1*α*
JNK: c-Jun N-terminal kinase
CaMKII: Calmodulin-dependent protein kinase II
Fis1: Mitochondrial fission protein 1
PKC: Protein kinase C
eNOS: Endothelial nitric oxide synthase
VEGF: Vascular endothelial growth factor
TLR-4: Toll-like receptor-4
NF-*κ*B: Nuclear factor kappa-B
TLR-6: Toll-like receptor-6
NLRP-3: NOD-like receptor protein-3
AIM2: Absent in melanoma 2
IL-1*β*: Interleukin-1*β*
IL-18: Interleukin-18.
